# Conductive Hydrogel Motion Sensor with Low-Temperature Stability for Winter Sports and Sensing Rescue

**DOI:** 10.3390/polym17101365

**Published:** 2025-05-16

**Authors:** Wei Li, Yang Ming, Libing Yang, Yimeng Ni, Yu Chen, Weidong Xu, Lefei Li, Chan Zheng, Wanyang Lin

**Affiliations:** 1 College of Materials Science and Engineering, Fujian University of Technology, Fuzhou 350118, China; mingyang5893@163.com (Y.M.); lbyang4518@163.com (L.Y.); chenyu20001204@163.com (Y.C.); weidong20000217@163.com (W.X.); zcfjut@163.com (C.Z.); 2 College of Chemical Engineering, Fuzhou University, Fuzhou 350116, China; niyimeng@uhrs.edu.cn; 3 School of Information and Smart Transportation, Fujian Chuanzheng Communications College, Fuzhou 350007, China; linwanyang@fjcpc.edu.cn

**Keywords:** conductive hydrogel, anti-freezing, phytic acid, sports sensing

## Abstract

Hydrogels with conductive properties hold significant promise in the realm of flexible electronics, owing to their pliability, outstanding conductivity, and diverse functionalities. Nevertheless, the majority of conductive hydrogels are prone to being brittle and easily damaged; as such, they are not adapt to cold environments, which seriously hinders their practical applications. Therefore, hydrogels that possess both conductivity and anti-freezing, as well as moisturizing, capabilities have garnered considerable interest, and these hydrogels can work stably in harsh environments. Phytic acid (PA), which mainly exists in plant seeds, is a kind of natural compound widely existing in nature that can be recycled; it provides electrical conductivity and anti-freezing to hydrogels. Here, a highly conductive hydrogel with excellent anti-freezing and moisturizing capabilities was prepared by incorporating PA into a polyacrylamide/gelatin hydrogel. The incorporation of PA endowed the hydrogel with an excellent conductivity of 5.8 S·cm^−1^. In addition, robust hydrogen bonding was formed between water and phytic acid molecules, and the hydrogel demonstrated remarkable anti-freezing and water retention. On this basis, hydrogels can be used for human winter sports sensing and low-temperature environmental alarm devices to provide faster rescue. This study provides a novel method for the development of hydrogels with low-temperature stability, and provides a revelation for the application of anti-freezing hydrogels in icy and snowy environments.

## 1. Introduction

As science and technology advance rapidly, flexible wearable sensors have demonstrated significant promise across various domains, notably in human–computer interactions [1,2], artificial intelligence [3,4], personalized healthcare [5,6,7], and electronic skin technologies [8,9,10]. Compared to traditional mechanical sensors, these sensors have excellent flexibility and sensitivity, so they are more suitable for use on the human body. Currently, researchers have effectively developed wearable, flexible electronic devices using an array of pliable materials as substrates, such as textiles [11,12,13], foams [14,15,16], aerogel materials [17,18,19], and hydrogels [20,21,22]. Hydrogels are a type of commonly used soft material, constructed from a three-dimensional, elastic, crosslinked polymer network and integrated with a large amount of water. Their unique structure endows them with many excellent properties, including excellent hydrophilicity, outstanding flexibility, significant stretchability, adjustable mechanical properties, and excellent biocompatibility [23,24]. These characteristics give hydrogels a significant advantage in mimicking the structure of natural soft tissues, making them an ideal material for manufacturing flexible wearable sensors [25,26,27].

Conductive hydrogels have a soft texture, high stretchability and high sensing sensitivity, so they are ideal substrates for flexible wearable electronic products. Conductive hydrogels can be prepared by incorporating conductive materials into the hydrogel matrix, giving them excellent electrical properties. Although hydrogel sensors are favored for their superior flexibility and sensing sensitivity, their performance under harsh environmental conditions remains challenging to maintain. For example, in cold environments, hydrogels are prone to freezing, which may cause damage to their structures, affecting their mechanical properties and electrical conductivity. In addition, hydrogels may dry and harden due to the gradual evaporation of water, which seriously affects their properties [28,29]. Thus far, three effective methods have been discovered to enhance hydrogels’ low-temperature tolerance. Salts like CaCl_2_, LiCl, and ZnCl_2_ can be added to boost conductivity and environmental stability [30]. Ionic liquids can also be incorporated to maintain functionality at low temperatures and after long-term storage [31]. Another approach is preparing organohydrogels by integrating organic solvents such as glycerol, ethylene glycol, sorbitol, and dimethyl sulfoxide [32,33]. However, these methods have drawbacks. Salt-doped hydrogels may have poor water-holding ability. Ionic liquid-based hydrogels face high costs issues [34]. In addition, as for ionic conductive organo-hydrogels, the integration of organic solvents will increase the system viscosity and lead to an enhancement of the resistance of ionic migration, thus significantly weakening ionic conductivity [35]. Thus, enhancing the mechanical toughness and electrical conductivity of hydrogels in low-temperature environments remains a significant challenge.

Here, based on the hydrophobic association design, we designed a conductive hydrogel with excellent mechanical properties, a wide strain-sensing range, low-temperature stability and easy adhesion to multiple substrates, which can be used as a low-temperature wearable strain sensor. The conductive hydrogel consists of gelatin, hydrophilic acrylamide and hydrophobic, crosslinked copolymers of octadecyl methacrylate (C_18_), sodium dodecyl sulfate (SDS), NaCl and PA. Due to the ionization of PA, the hydrogel also has an electrical conductivity of 5.8 S·cm^−1^. In addition, benefiting from the strong hydrogen bond interaction between PA and water molecules, the hydrogel showed good low-temperature stability, maintaining a 1260% tensile ratio and 3.68 S·cm^−1^ high conductivity even at −40 °C; it exhibited a wide strain-sensing range (0–900%) and high strain sensitivity (2.62). Based on this performance, the hydrogel can achieve efficient sensing in snowy and icy environments, protecting the lives and safety of winter athletes or expedition personnel.

## 2. Experimental Design

### 2.1. Materials

Sodium dodecyl sulfate (SDS, 98.5%), sodium chloride (NaCl, 99.5%) and gelatin were purchased from Aladdin Biochemical Technology Co (Shanghai, China). Phytic acid solution (50%), acrylamide (AAm, 99%) and octadecyl methacrylate (C_18_, 96%) were purchased from Shanghai McLean Biochemical Co (Shanghai, China). Ammonium persulfate (APS, 98%) and N,N′-methylene bis(acrylamide) were purchased from Sigma-Aldrich (Shanghai, China).

### 2.2. Preparation of Gelatin-SDS-NaCl-C_18_-PAAm-PA Hydrogels

Aqueous gelatin solutions of different concentrations were added together with 10 mL phytic acid solution; then, 0.8 g SDS, 0.32 g NaCl, and 100 μL C_18_ were added and stirred at 60 °C for 60 min. Then, 2 g AAm was added and stirring was continued for 30 min. Once cooled down, 0.01 g APS (initiator) and 2.0 mg MBAA (crosslinking agent) were added. Ultimately, the solution was poured into a mold and subjected to initiation at 60 °C for 1 h to yield gelatin-SDS-NaCl-C_18_-PAAm-PA hydrogels.

### 2.3. Measurements of Mechanical Properties

A tensile tester (UTM2102, 2018, Shenzhen Suns Technology Stock Co., Ltd., Shenzhen, China) was utilized to test the tensile and compressive properties of the hydrogels. In the tensile tests, hydrogels molded into a dumbbell shape (75 mm × 4 mm × 2 mm) were subjected to elongation at a rate of 100 mm·min^−1^. For the compression tests, cylindrical-shaped hydrogels (20 mm × 20 mm) were deformed under pressure at a velocity of 100 mm·min^−1^.

### 2.4. Measurements of Electrical Properties

The resistivity of the hydrogels was measured with the aid of a four-probe resistivity tester (Helpass, 2018, HPS2663, Changzhou, China). The conductivity (*σ*) was determined by the following equation:

(1) $$\sigma =\frac{1}{\rho }$$

where *ρ* is the resistivity of the hydrogel. To evaluate the sensing performance, the resistance of the hydrogels was tested at different strains and stretching rates, while the relative resistance changes were continuously monitored using a digital source meter (Tong hui, TH2832, 2020, Changzhou, China). The strain sensitivity of the hydrogel was characterized by its strain factor (GF), which was determined by the following equation:

(2) $$GF=\frac{\left(R-R_{0}\right)}{R_{0}\varepsilon }$$

In the equation, *R* and *R*_0_ represent the measured resistance value and the initial resistance of the hydrogel, respectively. ε signifies the strain applied. The relative change in resistance (Δ*R*/*R*_0_) was calculated as the ratio of the alteration in resistance (Δ*R*) during deformation relative to the initial resistance (*R*_0_) at 0% strain.

The low-temperature sensing performance test involves encapsulating the hydrogel and placing it in an ultra-low-temperature freezer set at −40 °C. Subsequently, the hydrogel is connected to a digital bridge via wires for testing.

### 2.5. Differential Scanning Calorimetry Analysis

The frost resistance of hydrogels was characterized by differential scanning calorimetry (DSC25, TA Instruments, 2016, New Castle, DE, USA) in a nitrogen-protected atmosphere. The specific test procedure is as follows: (1) cool down from 25 °C to −80 °C at a rate of 10 °C/min; (2) isothermal at −80 °C for 5 min to eliminate the thermal history; and (3) heat up to 25 °C at a rate of 5 °C/min. When preparing the sample, the hydrogel was cut into discs with a diameter of 2 mm and a thickness of 2 mm and placed in a standard aluminum crucible.

### 2.6. Fourier Transform Infrared Spectroscopy Measurements

The hydrogel was dried and processed into thin sheets of 20 mm × 10 mm to analyze the chemical structure by Fourier transform infrared spectroscopy (Thermo, Nicolet iS20, 2019, Boston, MA, USA). Select the ATR mode (with the crystal type being ZnSe), choose the transmittance mode for the test mode, and set the spectral range between 600 and 4000 cm^−1^.

### 2.7. Drying-Resistance Capabilities

Cylindrical hydrogels (20 mm × 20 mm) with and without PA were prepared and exposed to ambient conditions (25 °C, 50% humidity) for a duration of 15 days. The mass of the hydrogels was documented at one-day intervals. The remaining mass ratio was determined using the following formula:

Residual mass ratio (%) = *M_t_*/*M_i_* × 100% (3)

where *M_t_* signifies the original mass of the hydrogels, and *M_i_* indicates the mass of the hydrogels following various storage durations.

## 3. Results and Discussion

### 3.1. Preparation of Gel-Mic-PAAm-PA Hydrogel

**Figure 1:**
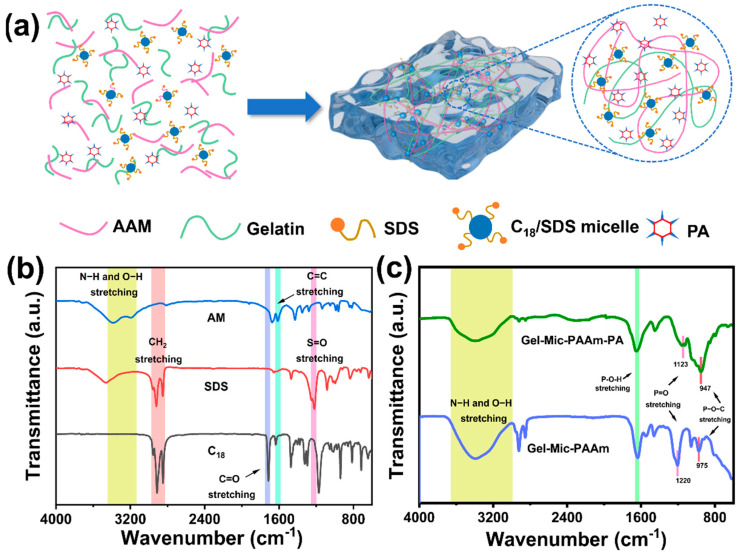
(**a**) The fabrication steps of the Gel-Mic-PAAm-PA hydrogel are illustrated; (**b**) FTIR analysis of AM, SDS and C_18_; (**c**) FTIR analysis comparing the Gel-Mic-PAAm-PA hydrogel with the Gel-Mic-PAAm hydrogel.

In this paper, a kind of Gel-Mic-PAAm-PA hydrogel was developed by the simple one pot method. The Gel-Mic-PAAm-PA hydrogel has excellent low-temperature conductivity, anti-freezing, and self-adhesive properties. Figure 1 shows the preparation process of this hydrogel. Firstly, PA was added to the aqueous gelatin solution. Secondly, SDS and C_18_ were added and stirred to obtain a uniform Gel-Mic-PAAm-PA mixture. Finally, the hydrogel was obtained by heat triggering.

In the reaction process, the surfactant SDS interacts with the hydrophobic alkyl chains of the C_18_ units to form micelles, which serve as dynamic hydrophobic association (HA) crosslinking points that connect with hydrophilic chains to create hydrophobic bonds and hydrogels. A combination of gelatin and SDS is incorporated into the hydrogel to form a composite surfactant system. Gelatin features both hydrophilic and hydrophobic regions, the gelatin’s hydrophobic segments attach to the droplet surfaces, and its hydrophilic segments project into the aqueous phase, preventing the droplets from merging. This prevents the coalescence of droplets and effectively stabilizes the hydrophobic parts, and the synergistic effect also improves the hydrogel’s structural strength and durability.

Moreover, the substantial release of free H^+^ ions from PA confers significant electrical conductivity to the hydrogel. Consequently, these conductive hydrogels are suitable substrates for wearable strain sensors. In addition, the addition of phytic acid can effectively improve the anti-drying and anti-freezing properties of hydrogel. Consequently, the Gel-Mic-PAAm-PA hydrogel demonstrates exceptional low-temperature stability and moisture retention.

Here, FTIR spectra of SDS, C_18_, and AM were tested to further explain the chemical reaction process and crosslinking behavior of the hydrogel. As shown in Figure 1b, the peaks at 1220 cm^−1^, 2851 cm^−1^, and 2919 cm^−1^ correspond to the S=O stretching vibration, symmetric stretching vibration, and asymmetric stretching of the -CH_2_- bonds in SDS, respectively. The C=C stretching vibrations of C_18_ and AM correspond to the peaks at 1635 cm^−1^ and 1613 cm^−1^, and the crest at 1713 cm^−1^ corresponds to the C=O stretching vibrations in them. In contrast, the crosslinked Gel-Mic-PAAm hydrogel spectra showed different characteristic peaks at 1613 cm^−1^ (AM), 1220 cm^−1^ (SDS) and 1713 cm^−1^ (C_18_) (Figure 1c). In addition, the broad peak at 3350 cm^−1^ is primarily attributed to the stretching vibrations of the N-H in the -NH_2_ groups and the O-H within the molecular structure. The disappearance of the C=C vibrational signal from AM and C_18_ indicates that these two compounds have formed a crosslinked hydrogel network through chemical bonding. Meanwhile, the S=O vibration frequency of SDS remains unchanged, suggesting that the SDS micelles are evenly dispersed throughout the entire hydrogel matrix [36]. The characteristic peaks of the Gel-Mic-PAAm-PA hydrogel at 947 cm^−1^, 1647 cm^−1^, and 1123 cm^−1^ are indicative of the stretching vibrations of the P-O-C, P-O-H and P=O bonds, respectively, and the successful incorporation of PA is confirmed. The introduction of PA has supplied a significant number of hydroxyl groups (O-H), which engage in the formation of hydrogen bonds. This interaction leads to the broadening of the characteristic peaks in 2971–3730 cm^−1^.

### 3.2. Mechanical Properties of the Hydrogel

**Figure 2:**
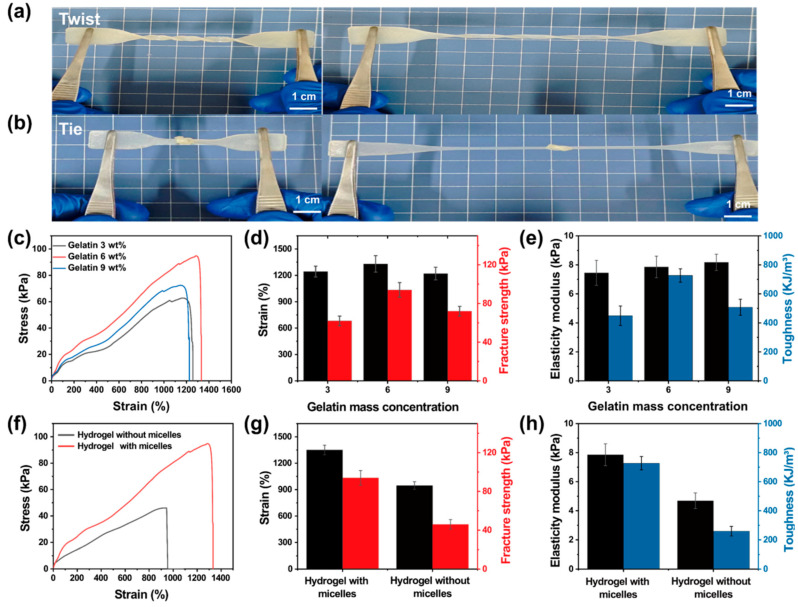
Schematic illustration of Gel-Mic-PAAm-PA hydrogels tensile test (**a**) during twisting and (**b**) knotting; (**c**) the stress–strain curves, (**d**) strain and fracture strength, and (**e**) elastic modulus and toughness of Gel-Mic-PAAm-PA hydrogels with the impact of varying gelatin concentrations; (**f**) the stress–strain curves, (**g**) strain and fracture strength, and (**h**) elastic modulus and toughness of hydrogels with/without micelles.

The outstanding mechanical characteristics of hydrogels are the key factors in ensuring long service lives for strain sensors. As shown in Figure 2a,b and Figure S1, the hydrogel can still be stretched to more than three times its initial length, even when twisted and knotted. The hydrogel was also resistant to tweezer bursting and bending. These results indicate that hydrogels have excellent mechanical properties.

The impact of varying gelatin concentrations on the mechanical properties of the Gel-Mic-PAAm-PA hydrogel was investigated, as shown in Figure 2c–e. The results revealed that the hydrogel with a gelatin concentration of 6 wt% demonstrates the best mechanical properties (with a strain of 1330%, a breaking strength of 94 kPa, an elastic modulus of 7.9 kPa, and a toughness of 727 kJ·m^−3^). Gelatin can form robust hydrogen bonds with the AAm network, but an overabundance of gelatin can reduce the interaction between the two networks. This reduction is possibly due to the self-assembly of gelatin macromolecules and the deterioration of hydrogen bond connections between the self-assembled gelatin and PAAm [37].

Secondly, micelles were introduced into the hydrogels to promote hydrophobic interactions and improve the mechanical properties. As shown in Figure 2f–h, the tensile strain of hydrogel effectively increases when micelles are introduced; the breaking strength was increased from 46 kPa to 94 kPa and the elongation increased from 942% to 1320%. This was attributed to the copolymerization of C_18_ with AAm in SDS aqueous solution, and the addition of NaCl promoted the formation of larger SDS micelles. The hydrophobic sequence was introduced into the hydrophilic polymer chain, generating a dynamic hydrophobic association between the hydrophobic region of the polymer chain and the surfactant micelle through the physical crosslinking of the resulting hydrogel [38,39].

### 3.3. Anti-Freezing and Anti-Drying Performance Test

Due to the presence of a high amount of water, traditional conductive hydrogels are prone to crystallization in sub-zero environments, resulting in decreased mechanical properties and a loss of electrical conductivity.

**Figure 3:**
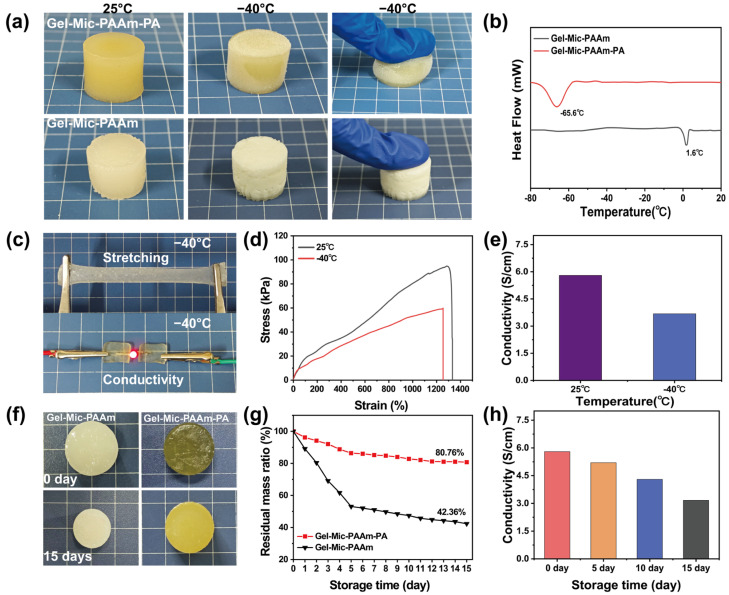
(**a**) Hydrogels prior to and following 24 h storage at −40 °C; (**b**) DSC of hydrogels; (**c**) hydrogels that are both stretchable and conductive at −40 °C; (**d**) stretching curves and (**e**) conductivity of Gel-Mic-PAAm-PA hydrogels at 25/−40 °C; (**f**) photographs of hydrogels taken before and following 15 days of exposure in an unsealed environment; (**g**) curves depicting the remaining mass ratio of hydrogels; (**h**) conductivity of Gel-Mic-PAAm-PA hydrogels after different preservation times.

Here, PA was introduced as an electrolyte to obtain an anti-freezing and highly conductive hydrogel. As depicted in Figure 3a, the hydrogels with or without PA were placed at −40 °C for 24 h, and the hydrogels without PA were completely frozen and could not be compressed, while the hydrogels containing PA could still be compressed. Figure 3c,d show that the hydrogel spline was stretched after freezing; the maximum fracture stress of the hydrogel after freezing decreased (59 kPa) and the tensile rate decreased slightly (from 1330% to 1260%). In addition, the conductivity of the hydrogel is 3.68 S·m^−1^, higher than other conductive hydrogels (Table 1), and the hydrogel can be used as a conductor to light a diode bulb. The freezing temperature of the anti-freezing hydrogels was determined by using differential scanning calorimetry (DSC); the Gel-Mic-PAAm hydrogel without PA showed an exothermic peak at 1.6 °C, while Gel-Mic-PAAm-PA exhibited an extremely low freezing point at −65.6 °C (Figure 3b). Experiments showed that the introduction of PA effectively improved the anti-freeze ability of the hydrogel, and the prepared hydrogel had the advantages of low-temperature conductivity (Figure 3e) and excellent mechanical properties.

When exposed to an open environment, the water within the hydrogel can evaporate quickly, significantly reducing its service life. Therefore, it is essential for the conductive hydrogel to possess resistance to drying. The Gel-Mic-PAAm-PA hydrogel with PA can still bend and stretch after 15 days of exposure in an open environment, and the tensile strain remains 1030% (Figure S7). However, the Gel-Mic-PAAm hydrogel without PA showed volume shrinkage (Figure 3f). Figure 3g illustrates the residual mass curves for hydrogels, both in the presence and absence of PA. After 15 days of exposure at room temperature, the weight of the Gel-Mic-PAAm hydrogel decreased by 57.64%, while the Gel-Mic-PAAm-PA had a weight retention rate of 80.76%. With the extension of retention time, the water in the gel is gradually lost, and the conductivity of the gel gradually decreases (Figure 3h). The hydrogel was not completely dried, so the conductivity could still reach 3.17 S·cm^−1^ after 15 days of retention.

**Figure 4:**
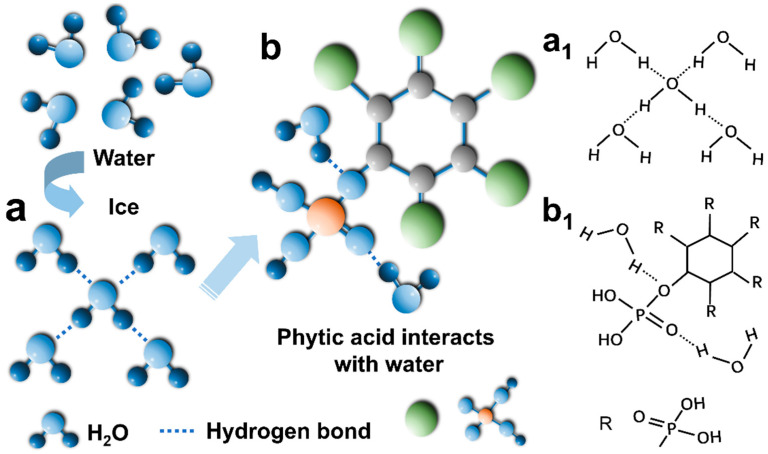
(**a**) The arrangement of water molecules after water freezes; (**b**) Phytic acid forms strong hydrogen bonds with water molecules.; (**a_1_**,**b_1_**) Molecular structure diagram.

Furthermore, the effect of phytic acid on the anti-freezing ability of hydrogels was studied. Figure 4a shows the arrangement of water molecules after water icing. When the water temperature is lower than 0 °C, the kinetic energy of water molecules is not enough to overcome the hydrogen bond, and the water molecules are arranged regularly. When phytic acid is added to the hydrogel, many highly electronegative oxygen atoms in phytic acid can act as hydrogen atom receptors and form strong hydrogen bonds with water molecules (Figure 4b); as such, hydrogen bonds cannot be generated between water molecules, thus inhibiting water icing. Infrared spectroscopy is the most effective and extensive method for studying hydrogen bonds [40]. As shown in Figure 1c, when PA was added to the hydrogel, the characteristic peaks of P=O and P-O-C redshifted from 1220 cm^−1^ to 1123 cm^−1^ and from 975 cm^−1^ to 947 cm^−1^, respectively, confirming the formation of strong hydrogen bonds between phytic acid and water molecules. The characteristic peak redshift of P=O is the most obvious, which may be because the oxygen atom is more electronegative and distant from other atoms, so it attracts H atom more strongly in water (Figure 4b_1_). In addition, the strong hydrogen bonds also inhibit crystallization and evaporation within the hydrogel [41,42,43].

**Table 1:** An evaluation of the low-temperature electrical conductivity of the Gel-Mic-PAAm-PA hydrogel in comparison to other documented anti-freezing hydrogels or organic hydrogels.

Anti-Freezing Agents	Conductive Components	Conductivity at 25 °C (S·cm^−1^)	Low-Temperature Conductivity (S·cm^−1^)	Ref.
Phytic acid	Phytic acid	5.8	3.68 (−40 °C)	This work
Glycerol/LiCl	LiCl	5.249	3.649 (−40 °C)	[44]
CNFs	Propylene carbonate	4.2	1.8 (−40 °C)	[45]
Glycerol	ANI	6.69	1.71 (−40 °C)	[46]
Ethylene glycol	AMIM-CL	0.48	0.45 (−20 °C)	[47]
LiCl	LiCl	8.99	2.58 (−20 °C)	[48]
DMSO	CMC-Na	2.1	1.7 (−40 °C)	[49]
Glycerol	^a^ LMNPs	1.2	0.55 (−20 °C)	[50]
^a^ VBIMBr	^a^ VBIMBr	1.303	0.849 (−20 °C)	[51]
KOH	KOH	10.5	1.81 (−20 °C)	[52]

^a^ LMNPs: liquid metal nanoparticles; VBIMBr: 1-butyl-3-vinylimidazolium bromide.

### 3.4. Motion Sensing of Gel-Mic-PAAm-PA Hydrogel

The outstanding mechanical characteristics ensure that flexible sensors can accurately detect a wide range of strains. As depicted in Figure S2a, the cyclic tensile loading is represented by a typical stress–strain curve. The pronounced hysteresis loops demonstrate substantial energy dissipation occurring throughout the cyclic loading process. Notably, as the strain increases, the size of these hysteresis loops in the loading and unloading curves expands. Figure S2b shows 10 consecutive loading cycles under 100% strain, with no recovery time between cycles. Because hydrogen bond breaking and electrostatic interactions dissipate massive energy, an obvious hysteresis curve appears in the first cycle; other than that, the curves of the other cycles are almost the same, showing remarkable fatigue resistance and excellent cyclic load fatigue resistance [53]. The hydrogel also displayed an indifference to the speed at which it was stretched, demonstrating its excellent elastic recovery ability (Figure S2c). Moreover, the shape of the hydrogel recovered rapidly after pressing with a finger (Figure S3), and the hydrogel was not damaged when the surface was cut with a knife (Figure S4).

**Figure 5:**
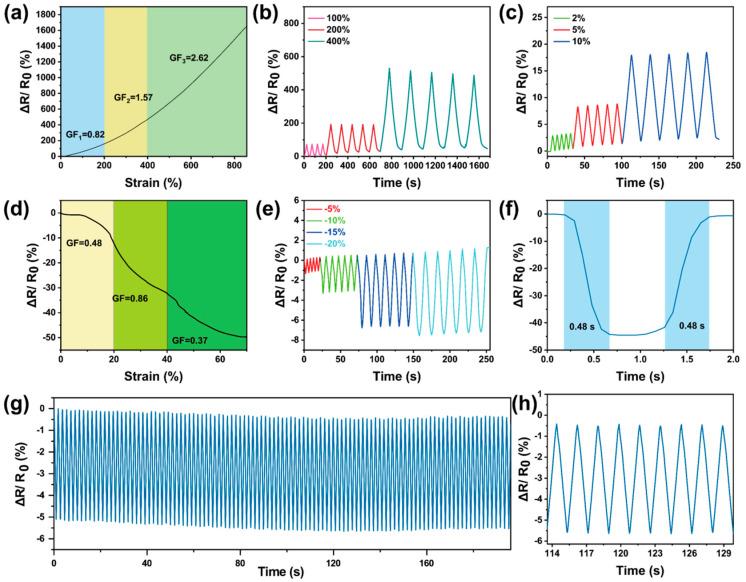
(**a**) Tensile strain coefficient of hydrogel; (**b**,**c**) relative resistance change in the hydrogel sensor at small and large strains; (**d**) compressive strain coefficient of hydrogel; (**e**) the change in relative resistance of hydrogel during repeated compression under different strains; (**f**) sensor response and recovery time at 50% strain; (**g**) hydrogel cyclic load test and (**h**) local magnification.

The Gel-Mic-PAAm-PA hydrogel has high electrical conductivity and mechanical flexibility, which means it can be used as a piezoresistive hydrogel strain sensor. The assembled conductive hydrogel strain sensor has a broad strain response, with a strain range of 0–900% (Figure 5a). During the stretching process, the free ion transport path in the hydrogel becomes longer and narrower, resulting in increased resistance, and the relative resistance (ΔR/R_0_) increases with the increase in the tensile strain [54]. The gauge factor (GF) is a key parameter for evaluating the performance of hydrogel-based strain sensors.

The GFs of the Gel-Mic-PAAm-PA conductive hydrogel in 0–200%, 200–400% and 400–900% strain ranges are 0.82, 1.57 and 2.62, respectively (Figure 4a). Figure 5b,c show that the hydrogel output cyclic signal was stable at small (2–10%) or large (100–400%) strains. In addition to its conducting, the hydrogel has a fast tensile response time (0.46 s) and recovery time (0.39 s) (Figure S5). The GF of conductive hydrogel compression sensitivity is presented in Figure 5d. Hydrogels also exhibit excellent strain-sensing reversibility under compression, as shown in Figure 5e. The time response is likewise a critical parameter for assessing the sensor’s performance. The hydrogel sensor has a fast response time (0.48 s) and recovery time (0.48 s) (Figure 5f). As shown in Figure 5g,h, the hydrogel sample was subjected to 100 compression/release tests at 10% strain. During the compression cycle test, there was little drift or hysteresis of the electrical signal. The Gel-Mic-PAAm-PA hydrogel also has certain adhesion properties, as shown in Figure S6a; it can be attached to plastic, iron, wood, rubber, glass, etc., showing excellent adhesion. Even at −40 °C, the hydrogel maintains adhesion (Figure S6b). This is crucial for maintaining the stability of the resistance output during the sensor’s operational monitoring.

This indicates that the hydrogel has the design conditions of the sensor, strain-sending reversibility, a stable signal output, and outstanding mechanical properties, as well as showing that the action response was sensitive.

### 3.5. Gel-Mic-PAAm-PA Hydrogel for Wearable Sensing Devices

**Figure 6:**
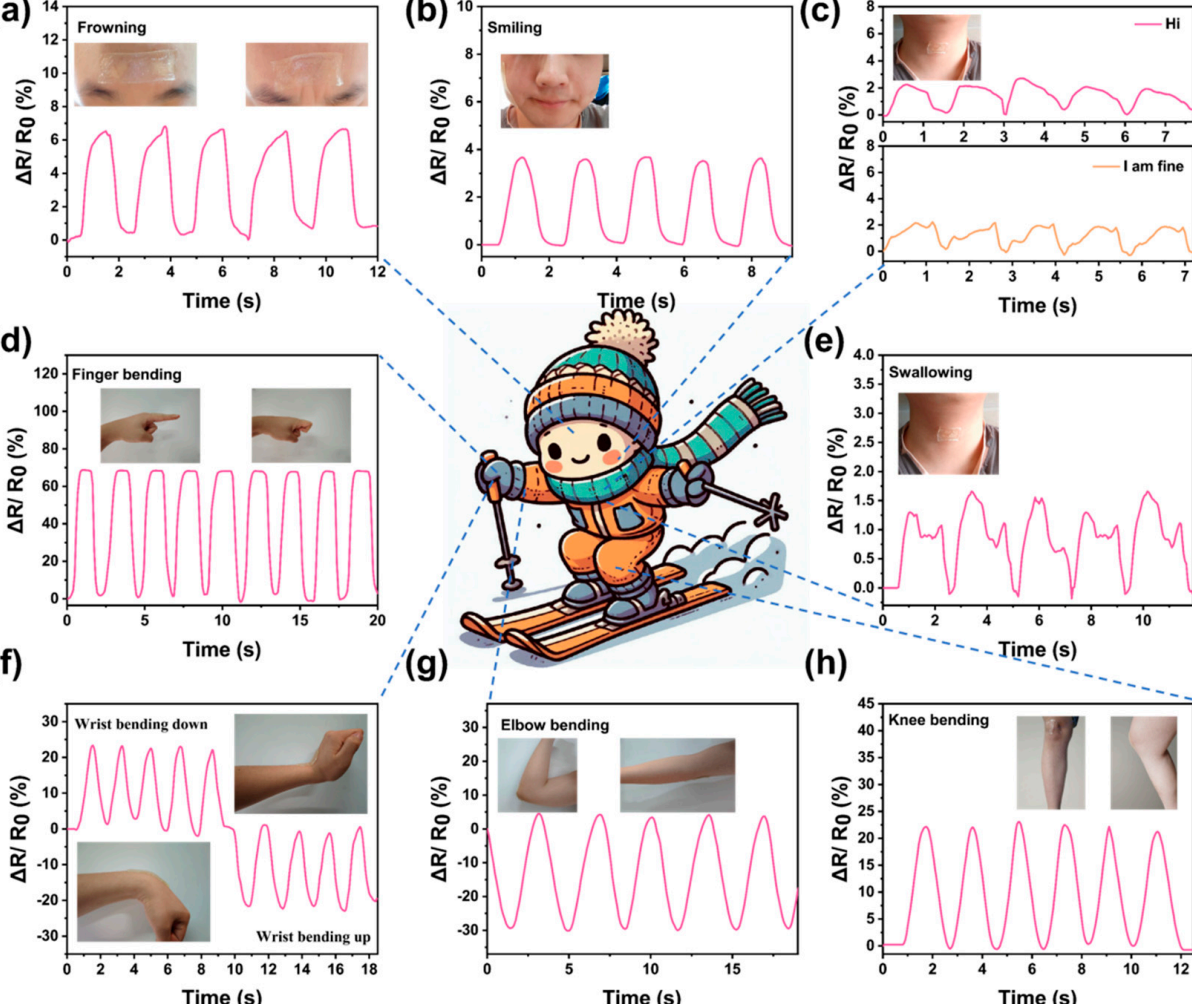
The Gel-Mic-PAAm-PA hydrogel sensor monitors human movements: (**a**) frowning; (**b**) smiling; (**c**) speaking; (**d**) finger bending; (**e**) swallowing; (**f**) wrist movements; (**g**) elbow movement; (**h**) legs.

It is worth noting that, due to its excellent frost resistance, the Gel-Mic-PAAm-PA hydrogel strain sensor can still exhibit superior mechanical strength and a broad sensing range, even under low-temperature conditions of −40 °C. This feature can be used for the multi-position motion detection of skiers; we affixed them to various human body joints for the purpose of tracking human motion. Figure 6f–h show the output resistance signals delivered by large movements when the hydrogel was applied to the knee, elbow, and back of the hand; Figure 6a–e show the resistance signals delivered by small movements when the hydrogel was applied to the finger, throat, and face. These signal cycles are stable and clear. As shown in Figure 6c, after applying the hydrogel to the throat, it can recognize the pronunciation of words, such as “Hi, I am fine, etc.”. The deformation of the hydrogel caused by this weak vibration can also be converted into a clear signal.

**Figure 7:**
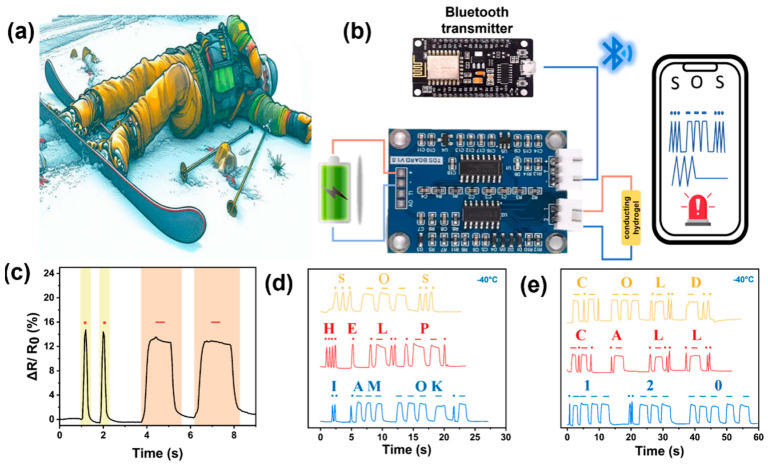
(**a**) Skiers waiting to be rescued; (**b**) components of the designed intelligent monitoring and alarm system. (**c**) The dot and dash signals produced by the hydrogel sensor at −40 °C; (**d**,**e**) encrypted messages transmitted using morse code.

As a prospective safety-monitoring solution, this study proposes a conductive hydrogel-based emergency alert system for skiers. As shown in Figure 7a,b, when skiers have an accident, hydrogels can be used to transmit alarm signals and quickly implement rescue. When the rapidly changing sensing signal suddenly disappears, it means that the person is in danger; at this time, the MCU sends a distress signal. In addition, skiers can use characteristic movements to signal for help. Morse Code was used to convert motion signals into electrical signals, which were then translated into distress calls, where the peak signal represents “·” and the sustained high-level signal represents “-” (Figure 7c). The transmission of complex information is realized through the combination of different and regular voltage signals. For example, the voltage signal is converted into a coded signal (“··· --- ···”, “·· · --- ----·-”, “·---- ··--- -----”) and translated into “SOS”, “I AM OK”, “120”, etc. (Figure 7d,e). Because the conductive hydrogel has the ability of accurate large and small strain monitoring, it is appropriate for large movement detection in skiing and small movement alarms after falling.

## 4. Conclusions

In this study, a Gel-Mic-PAAm-PA hydrogel based on polyacrylamide (PAAm) and gelatin was successfully synthesized. The resulting hydrogel demonstrated remarkable environmental stability, strong self-adhesive capability, outstanding freeze resistance, and excellent electrical conductivity. Leveraging the robust hydrogen bonding interaction between PA and water molecules, the crystallization temperature of the hydrogel was markedly decreased, and the resistance to drying was greatly improved, ensuring its stability at low temperatures and long-term preservation. The hydrogel has an electrical conductivity of 5.8 S·cm^−1^ at normal temperature, and even at a low temperature of −40 °C, the Gel-Mic-PAAm-PA hydrogel still has a tensile ratio of 1260% and an electrical conductivity of 3.68 S·cm^−1^. Its mass retention rate was as high as 80.76% after 15 days in the natural environment. In addition, Gel-Mic-PAAm-PA hydrogels exhibit extremely high strain sensitivity (GF = 2.62) and a wide strain-response range (−20–900%). This gives the hydrogel the potential to act as a wearable flexible sensor in monitoring a range of human motions (such as wrist bending, gesture changes, facial expressions and vocalizations) with fast responses and recognitions of both large and small movements. It is worth noting that the hydrogel has low-temperature stability and sensitive sensing ability, making it an inspiration for sensors and signal output in low-temperature environments.

## Data Availability

Data are contained within the article and Supplementary Materials. Further inquiries can be directed to the corresponding author.

## Supplementary Materials

The following supporting information can be downloaded at https://www.mdpi.com/article/10.3390/polym17101365/s1, Figure S1: The Gel-Mic-PAAm-PA hydrogels (Gel 6 wt%) resists tweezer bursting and bending; Figure S2: (a) the performance under different tensile strains; (b) 10 loading and unloading cycles at 200% tensile strain; (c) the stress-strain curves of the hydrogel at different stretching rates; Figure S3: Hydrogel before and after pressing; Figure S4: The Gel-Mic-PAAm-PA hydrogels (Gel 6 wt%) resisted the cut with a blade; Figure S5: Response and recovery time of Gel-Mic-PAAm-PA hydrogel strain sensor; Figure S6: (a-b) Demonstrates the excellent adhesion of Gel-Mic-PAAm-PA hydrogel to a wide range of organic and inorganic materials, including iron, wood, rubber, glass, and PTFE, and the maintenance of adhesion at low temperatures; Figure S7: Comparison of tensile curves of hydrogels after 15 days of storage in an open environment.
