# Supplementary material for: Conductive Hydrogel Motion Sensor with Low-Temperature Stability for Winter Sports and Sensing Rescue
Source: Polymers (Basel). 2025 May 16;17(10):1365. doi: 10.3390/polym17101365 (PMC12115171; doi:10.3390/polym17101365)
Supplement: Supplementary file 1 [file polymers-17-01365-s001.zip › polymers-3621125-supplementary.pdf]

Supporting Information

Low Temperature Stability Conductive Hydrogel Motion Sensor for Winter Sports and Sensing Rescue

Low Temperature Stability Conductive Hydrogel Motion Sensor for Winter Sports and Sensing Rescue

Wei Li[a *], Yang Ming[a], Libing Yang[a,b], Yimeng Ni[b], Yu Chen[a], Weidong Xu[a], Lefei Li[a], Chan Zheng[a], Wanyang Lin[c]

[a] *College of Materials Science and Engineering, Fujian University of Technology, Fuzhou 350118, PR China*

[b] *College of Chemical Engineering, Fuzhou University, Fuzhou 350116, PR China*

[c] *School of Information and Smart Transportation, Fujian Chuanzheng Communications College, Fuzhou 350007, PR China*

**Corresponding author. liwei@ fjut.edu.cn (W. Li)*

This PDF file includes:

Supplementary Figs. 1 to 7.

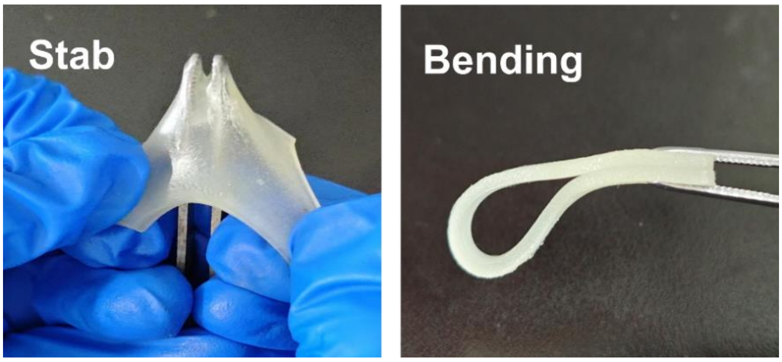

Figure S1. The Gel-Mic-PAAm-PA hydrogels (Gel 6 wt%) resists tweezer bursting and

bending.

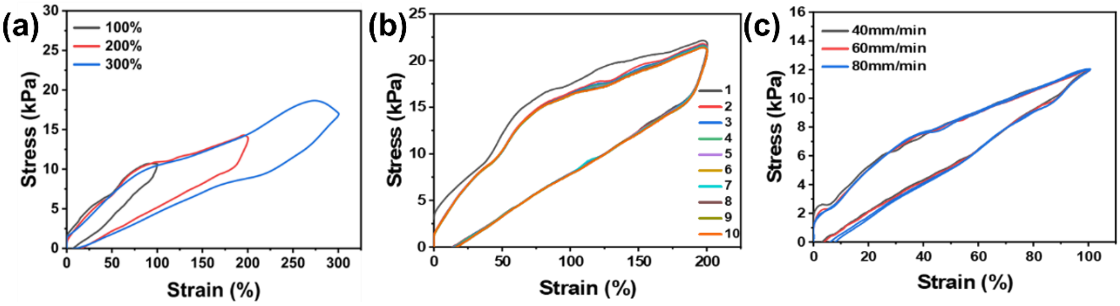

Figure S2. (a) the performance under different tensile strains; (b) 10 loading and unloading cycles at 200% tensile strain; (c) the stress-strain curves of the hydrogel at different stretching rates.

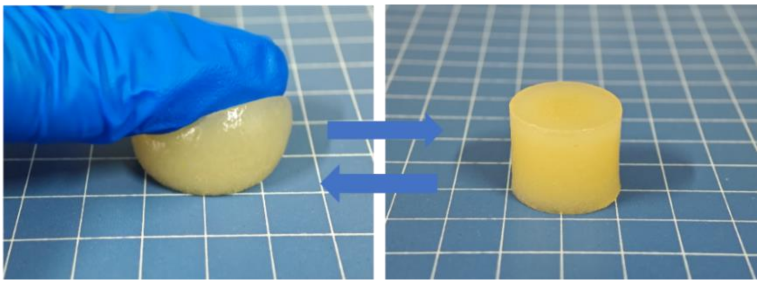

Figure S3. Hydrogel before and after pressing.

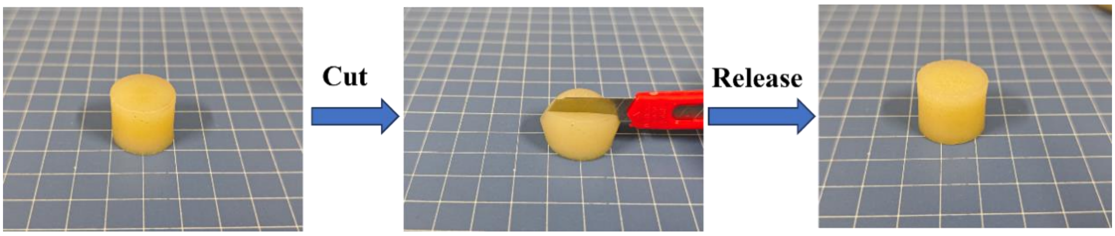

Figure S4. The Gel-Mic-PAAm-PA hydrogels (Gel 6 wt%) resisted the cut with a blade.

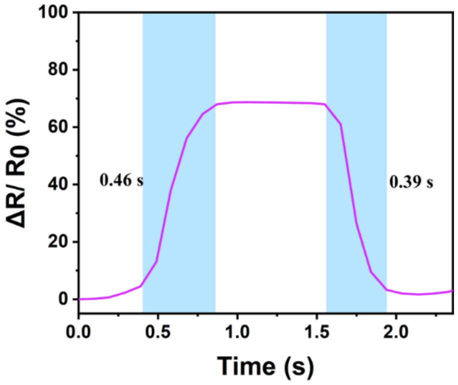

Figure S5. Response and recovery time of Gel-Mic-PAAm-PA hydrogel strain sensor.

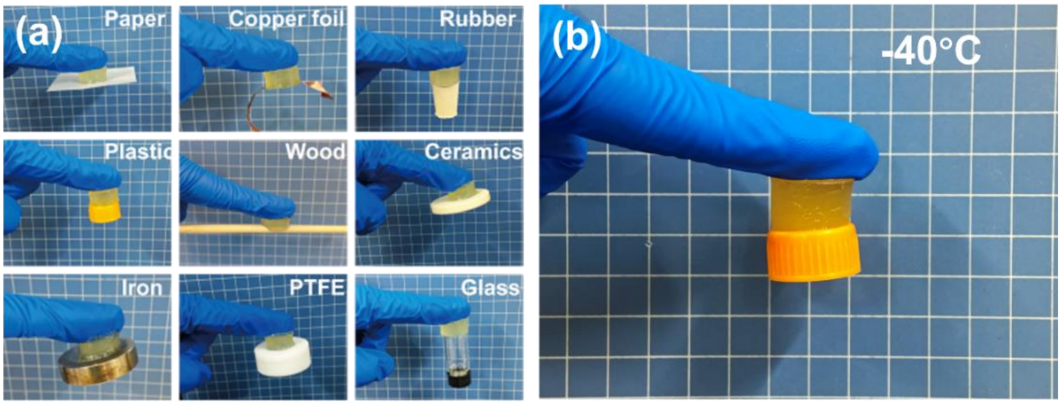

Figure S6. (a-b) Demonstrates the excellent adhesion of Gel-Mic-PAAm-PA hydrogel to a wide range of organic and inorganic materials, including iron, wood, rubber, glass, and PTFE, and the maintenance of adhesion at low temperatures.

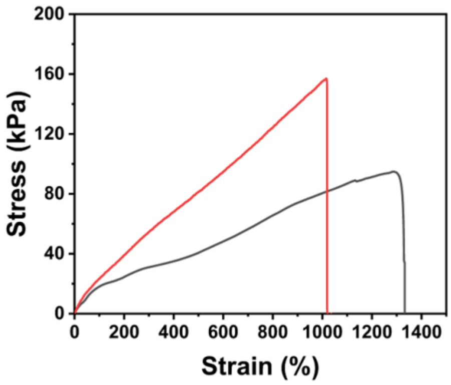

Figure S7. Comparison of tensile curves of hydrogels after 15 days of storage in an open environment.
